# Author Correction: A Mathematical Model for Vibration Behavior Analysis of DNA and Using a Resonant Frequency of DNA for Genome Engineering

**DOI:** 10.1038/s41598-021-97512-z

**Published:** 2021-08-31

**Authors:** Mobin Marvi, Majid Ghadiri

**Affiliations:** Faculty of Engineering, Department of Mechanics, Imam Khomeini International University, P.O. Box 34149-16818, Qazvin, Iran

Correction to: *Scientific Reports* 10.1038/s41598-020-60105-3, published online 26 February 2020

The original version of this Article contained a typographical error in Table 9, where the value of the First mode in “Number of Nucleobases”, “30” was incorrect. The correct and incorrect values appear below.

Incorrect:

**Table:** 

Number of Nucleobases	Number of turn of helix	Natural frequency (*Hz*)		
		First mode	Second mode	Third mode
30	3	5.2032 * 10^8^	1.1533 * 10^9^	1.8835 * 10^9^

Correct:

**Table:** 

Number of Nucleobases	Number of turn of helix	Natural frequency (*Hz*)		
		First mode	Second mode	Third mode
30	3	8.2032 * 10^8^	1.1533 * 10^9^	1.8835 * 10^9^

The original Article has been corrected.

